# Cutaneous presentation of disseminated cytomegalovirus infection in a non-transplant patient with hematological malignancy: A case report

**DOI:** 10.1097/MD.0000000000028721

**Published:** 2022-02-04

**Authors:** Hung-Chuan Yu, Wang-Da Liu, Po-Hsien Kuo, Chien-Chin Lin, Un-In Wu

**Affiliations:** a Department of Internal Medicine, National Taiwan University Hospital and National Taiwan University College of Medicine, Taipei, Taiwan; b Department of Medicine, National Taiwan University Cancer Center, Taipei, Taiwan; c Department of Internal Medicine, National Taiwan University Hospital Biomedical Park Hospital, Hsinchu, Taiwan; d Department of Laboratory Medicine, National Taiwan University Hospital, Taipei, Taiwan.

**Keywords:** case report, cellulitis, cytomegalovirus infection, hematological malignancy, lymphoma

## Abstract

**Rationale:**

Cytomegalovirus (CMV) disease is relatively uncommon in nontransplant hematological patients. Moreover, cutaneous manifestations of CMV diseases have scarcely been reported and are probably under-recognized.

**Patient concerns:**

We describe a patient with large B-cell lymphoma who developed a band-form, erythematous lesion over his left abdomen soon after the second course of rituximab, cyclophosphamide, doxorubicin, vincristine, prednisolone chemotherapy.

**Diagnoses:**

The lesion was initially mistaken for bacterial cellulitis or herpes zoster and was histologically confirmed as cutaneous CMV infection. Subsequent work-up also detected CMV viremia and the presence of CMV meningoencephalitis.

**Interventions:**

The patient was treated with ganciclovir plus CMV immune globulin followed by foscarnet.

**Outcomes:**

Although the patient's cutaneous lesion resolved, his cognitive impairment did not recover, and he developed a fatal multi-organ failure 1 month later.

**Lessons:**

Cutaneous CMV disease can herald multisystem involvement and an unfavorable prognosis in immunocompromised hosts. It should be ruled out with biopsy in patients with hematological malignancy who have cutaneous lesions refractory to antibacterial therapy.

## 1 Introduction

Cytomegalovirus (CMV) disease is generally associated with significant morbidity and mortality among immunocompromised patients. Common end-organ manifestations include pneumonitis, gastrointestinal diseases, hepatitis, retinitis, nephritis and encephalitis.^[1]^ While symptoms can be non-specific in patients with severe illness, cutaneous manifestations of CMV infection have been scarcely reported.^[2–4]^ Here, we present a rare case of disseminated CMV infection mistaken for cellulitis at disease onset in a patient with large B-cell lymphoma.

## 2 Case presentation

A 74-year-old man with diabetes mellitus was diagnosed of large B-cell lymphoma with bone marrow involvement (Ann Arbor stage IV) and had an initial presentation of fever and leukopenia. Flow cytometry analysis of his bone marrow specimens showed a group of clonal B cells with positive expression of CD19+, CD20+, and surface kappa light chain restriction. Cytogenetics of the bone marrow cells revealed a complex karyotype. He was treated with rituximab, cyclophosphamide, doxorubicin, vincristine, prednisolone 1 month prior to this event. His constitutional symptoms including fever and general malaise recovered well after the first course of treatment.

**Figure 1:**
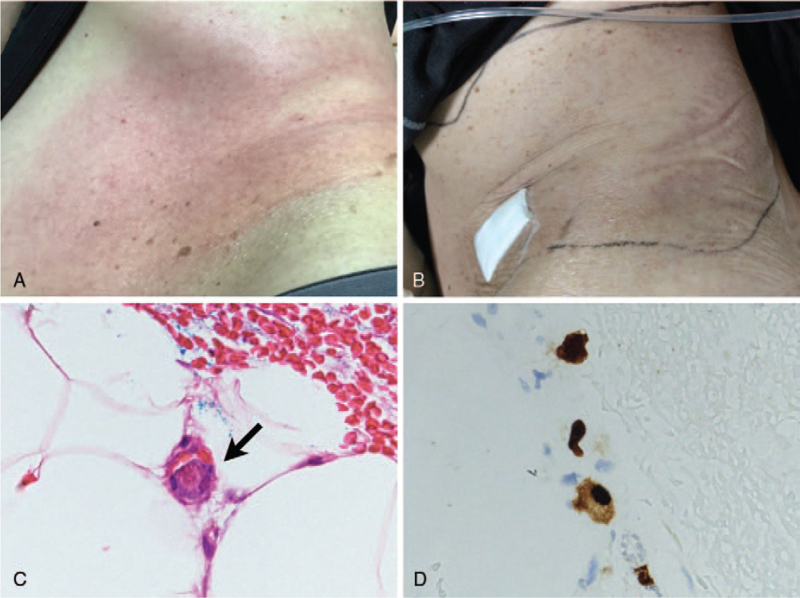
(A) Initial cutaneous manifestation over the left flank of the patient. (B) The lesion improved considerably 2 days after antiviral therapy. (C) Hematoxylin and eosin stain showing cytomegalic endothelial cells (arrow). (D) Immunohistochemistry staining showing inclusion-bearing cells typical of cytomegalovirus (“owl's-eye inclusions”).

The patient was then admitted for the second course of chemotherapy with rituximab, cyclophosphamide, doxorubicin, vincristine, prednisolone. On the second day after cessation of systemic steroids, fever and chills developed. On physical examination, a band-form, irregularly shaped erythematous patch with local heat and stinging pain emerged on his left abdomen (Fig. 1A). The patch rapidly extended to his left waist and lower back the following day in the absence of vesicles, bulla, desquamation or other superficial lesions. Blood examinations showed anemia (9.9 g/dL) and thrombocytopenia (53 × 10^3^/μL) which were comparable to his baseline levels. He had a reduced white blood cell count (2.8 × 10^3^/μL) with 94.2% neutrophils and 1% lymphocytes. Liver enzymes, renal function tests, C-reactive protein level (0.6 mg/dL) and other routine biochemistry tests were all within normal range or similar to his baseline levels. With a tentative diagnosis of shingles or cellulitis, the patient was treated with valacyclovir and a combination of broad-spectrum antibacterial agents. However, his fever persisted with increase in weakness, size and pain intensity of the skin lesion. A dermatologist was consulted and decided to perform a skin biopsy with the concern of lymphoma cutis or opportunistic infection. The histopathological examination revealed a sparse perivascular infiltrate of mononuclear cells with edema in the dermis and mild fat necrosis of the subcutaneous fat. A few endothelial cells showing markedly enlarged nuclei (Fig. 1C) were positive for CMV immunohistochemical staining (Fig. 1D). The infiltrating lymphocytes were all CD3+/CD20- T cells without any morphological evidence of lymphoma involvement.

Two days after the skin biopsy, the patient experienced a gradual onset of disorientation and dysphagia. Both computerized tomography and magnetic resonance imaging of the brain were unremarkable. Serum CMV viral load measured by real-time polymerase chain reaction (Roche AmpliPrep/TaqMan system) was found to be 4,710,000 copies/mL. His cerebrospinal fluid (CSF) was acellular with normal protein and glucose levels. However, CMV was detected via a nested multiplex polymerase chain reaction (BioFire/FilmArray system). A complete microbiological work-up did not identify any other microorganisms in the blood, CSF or biopsied specimens.

The cutaneous lesion vanished rapidly after administration of ganciclovir and anti-CMV IVIG therapy for 2 days (Fig. 1B). However, the patient's cognitive impairment progressed along with a slow decline in serum CMV viral load in the following 2 weeks. The antiviral regimen was subsequently changed to foscarnet, but his serum viral load did not decline to undetectable level. He was later admitted to the intensive care unit due to multi-organ failure and passed away on the 30th day after the diagnosis of disseminated CMV infection.

## 3 Discussion

While CMV is one of the most common viral pathogens causing various complications among recipients of hematopoietic stem cell transplantation, solid organ transplantation and HIV-infected individuals, CMV diseases are relatively uncommon in hematologic patients who do not undergo transplantation. Reports are limited to a few cases and postmortem studies. Patients with lymphoid hematologic malignancies appeared to have a higher risk of CMV reactivation (13.6%) than those with myeloid malignancies (3.9%).^[5]^ Poor performance status, high-dose steroid use, advanced stage of malignancies, ICU admission and previous exposure to fludarabine, bortezomib and monoclonal antibodies have also been reported to confer a high risk of CMV reactivation in such population.^[1,5–7]^ Our patient had had extremely low lymphocyte counts (less than 3% of total white blood cell) for at least one month and had received rituximab, steroids and chemotherapy, all of which contributed to his vulnerability. In a case series studying CMV infection among non-transplant hematological patients,^[8]^ a relationship was suggested between the use of rituximab/steroids and the development of CMV diseases in patients with non-Hodgkin's lymphoma. Notably, while rituximab is known to be associated with reactivation of hepatitis B virus and JC virus, there are cases describing its association with other unusual viral infections.^[9]^

CMV encephalitis is a serious complication of CMV infection. However, diagnosis is extremely difficult because CSF findings (e.g., pleocytosis and elevated protein) and radiographic changes may not be sufficiently sensitive or specific.^[10]^ The lack of efficacy in our patient may reflect a delay in the initiation of antiviral therapy (7 days after cutaneous manifestations) and poor blood-brain barrier penetration of antiviral agents.^[11]^

The first manifestation of CMV disease in our patient was cutaneous reactivation which is much less common than CMV encephalitis. While grouped vesicles in a dermatomal pattern suggest varicella zoster virus reactivation, cutaneous manifestations of CMV infection are variable and include ulcers, maculopapules, plaques, vesicles and nodules (Table 1).^[2–4]^ Overall, the most common presentation of cutaneous CMV infection among immunocompromised patients including hematopoietic stem cell transplantation recipients is anogenital ulcers, but extensive or disseminated cutaneous CMV infection following radiation therapy has also been described in patients with cutaneous T-cell lymphoma.^[12–13]^ Nevertheless, CMV infection presenting as cellulitis has scarcely been reported. This disease entity is probably under-recognized because it relies largely on histopathological examination.

**Table 1:** Summary of cutaneous CMV infection in the current and previous cases.

Reference	Dauden et al^[3]^	Choi et al^[2]^	Drozd et al^[4]^	Present case
Publication year	1987–1999	1999–2005	2007–2017	2019
No. of cases	17	9	57	1
Gender	Male (76.5%)	Male (77.8%)	Male (43.9%)	Male
Mean age, years	38.7 ± 9.0	41.2 ± 24.4	47.8 ± 24.4	74
Underlying disease (no. of cases)	HIV (17)	Non-HIV (9)	HIV (10)	Non-HIV (1)
		Lymphoma (2) Leukemia (2) Cirrhosis (2) Kidney transplantation (2) Aplastic anemia (1)	Non-HIV (47) Organ transplant (14) Bone marrow transplant (1) Malignancy (3) Autoimmune disease (10) Immunodeficiency (3) DIHS treated with steroid (4) ESRD (1) Urticaria (1) Substance abuse (1) No systemic disease (9)	Lymphoma (1)
Characteristic of skin lesion (no. of cases)	Ulcers (14) Perianal (10) Genital (3) Oral (3) Crusted vesicles (2) Ulcerated nodule (1)	Ulcers (6) Buttock (4) Penile shaft (1) Perineum (1) Nodule (2) Back (1) Anus (1) Maculopapular rash (1)	Ulcers (29) Perineal (16) Oral (4) Lower extremities (4) Facial and scalp (3) Dispersed (5) Papules/Macules (4) Purpura/petechia (3) Nodules (3) Ulcerated plaques (2) SJS/TEN (2) Erythema multiforme (1) Erythema and blister (1) Sclerodermoid change (1)	Band-form erythematous patch

CMV = cytomegalovirus, DIHS = drug-induced hypersensitivity syndrome, ESRD = end-stage renal disease, HIV = human immunodeficiency virus, SJS = Steven–Johnson syndrome, TEN = toxic epidermal necrolysis. Only cases of cutaneous CMV infection confirmed by histopathology were included.

## 4 Conclusions

Our case highlights that although rare, CMV diseases can occur in non-transplant hematological patients. Furthermore, disseminated CMV infection can manifest as cellulitis at the initial presentation. Since early recognition and initiation of antiviral therapy are key to improving outcomes of CMV diseases, and cutaneous presentation can herald a serious systemic infection in a profoundly immunosuppressed patient, an unexplained cutaneous lesion should prompt histopathological examination to direct effective therapy. Additionally, increased vigilance and aggressive studies are needed in hematological patients with altered mental status, particularly in the context of CMV viremia.

## Acknowledgments

The authors would like to thank Dr. Jau-Yu Liau, Department of Pathology, National Taiwan University Hospital, for providing photographs of the histopathology slides.

## Author contributions

**Conceptualization:** Hung-Chuan Yu, Po-Hsien Kuo.

**Data curation:** Hung-Chuan Yu, Wang-Da Liu.

**Supervision:** Un-In Wu.

**Writing – original draft:** Hung-Chuan Yu.

**Writing – review & editing:** Wang-Da Liu, Po-Hsien Kuo, Chien-Chin Lin, Un-In Wu.
